# Moral Aberration Due to Physical Irritants

**Published:** 1911-06-15

**Authors:** Henry S. Upson

**Affiliations:** Professor of Neurology in the Medical Department of Western Reserve University, Cleveland, O.


### MORAL ABERRATION DUE TO PHYSICAL IRRITANTS.

BY HENRY S. UPSON, M. D.
Professor of Neurology in the Medical Department of Western Reserve University, Cleveland, O.

A close study of insanity makes it increasingly apparent that it shows itself largely by disorders of conduct. While these often have their foundation in insane thoughts, this is by no means always the case. Insane conduct is so often anti-social that great numbers of the insane come into conflict with the authorities, and as the insane are largely removed from the possibility of reform by punishment, these offenders are especially prone to drift into the habitual criminal class, of which they form a large proportion. It becomes of the most urgent importance to recognize insane offenders as such, not for the purpose of establishing their innocence and freeing them which is the reverse of desirable for the good of society, but in order to prevent and cure the disease from which they suffer. It has long been known that the physical attributes of the insane and of habitual criminals are similar. Lavater noted many years ago the irregularity of the teeth of criminals and said that their mouths looked as if the teeth had been thrown into them from a distance. In the course of an investigation into impacted and otherwise diseased teeth in causing insanity I examined in the winter of 1908-09 eighteen of the younger inmates of the Cleveland Workhouse with a special view to impaction, and incidently a few of the older men for other lesions. Of the eighteen cases ranging from eighteen to twenty-five years of age, twelve showed multiple impactions. A number of these were operated on, and a part of the irritation relieved. It was difficult to obtain histories, and in most instances impossible to follow the case and give them adequate relief from the various lesions from which they suffered Such cases should be delt with early, before their lesions have become chronic and widespread, and aberrant conduct habitual. However each bit of wreckage saved from such a sea is so much clear gain, and three of these cases are sufficiently significant to warrant report at this time.

The first patient, a young man twenty-one years old, as a child was unusually bright truthful and honest. He was rather nervous, had tremors at times, and when two years old had a convulsion, but has had none since. He did well in study and deportment until he entered the high school; there he was rather mischievous, but remained throughout the course. Soon afterward, at the age of sixteen, he began to work for a business firm, and no fault was found with him by his employers, but he began to break into stores, committed robberies, and finally robbed a post-office. He was then sent to a reformatory, where he remained for two years, leaving at the age of nineteen. He then went to work in a box factory, and later made collections for a firm of attorneys. He finally held up a man on the street, and was sent to the house of correction for sixty days on the technical charge of assault and battery. During all the time after he left school his actions at home were peculiar, and apparently significant of a disordered mind. There were times when he was flighty and somewhat incoherent, and in addition he had periods of automatism, during which he would respond to questions, and was somewhat rigid. He had no recollection of these times. He was very peculiar in regard to his sleeping. He would throw the mattress on the floor and sleep on the springs of the bed even in cold winter weather. In addition to his criminal acts he has always tried to right the wrongs of other people, taking a hand whenever he thought anyone was abused, especially children. His impulses have been generous, and his robberies usually committed with the apparent motive of providing money for the sports of his comrades as well as himself. He has been knocked unconscious seven or eight times.

On examining the boy he was found to be strongly built, of good color and healthy appearance. He was unusually long limbed and short bodied, with a wide determined jaw, and pleasant face. Two brothers and two sisters are well mentally and physically, and the family is one of intelligence and high moral standards. An aunt on the fathers' side was insane, but there has been no other insanity or crime on either side of the family.

Skiagraphic examinations showed badly impacted wisdom teeth, abscesses at the roots of two of the lower molars, and an abscess at the root of one of the upper central incisors. The impacted and abscessed molar teeth were extracted in January, 1909. A month later James returned home and soon afterward obtained employment in a printing shop. Following the operation his mental state made a gradual improvement. His restlessness and irritability became less marked, and he has had no return of his spells of flightiness and automatism. With the object of removing all possible sources of irritation, so as to give him the best chance of recovery, he was circumcised early in June, and a moderate degree of astigmatism was found and corrected by glasses. The glasses have since been discarded without bad result. In the latter part of May the lateral incisor tooth, which showed evidence of absorption at the root, was extracted, and a marked abscess was found at the end of the root and another at the junction of the tooth and the artificial crown. The sequence of events following the extraction was unusually interesting. There was an acute exacerbation of symptoms. James was irritable, obstinate and contradictory so that sometimes it was impossible to carry on a conversation in his presence. He became once more restless at night and dissatisfied with his bed. These symptoms, however, were far from constituting a complete relapse. Improvement began again a week or two later, and was much more rapid than before the removal of the abscessed tooth. He is now quiet and industrious, has good self-control, and gives no evidence of either moral or mental aberration. He prefers out-door work, and the problem of a suitable employment still presents difficulties.

The mother at my request gives the following brief history, which seems to show a very early onset of the disease:

"My children were all considered very bright, but J. was by far the brightest. When four years old he could read and spell short words, tell the time correctly and play little tunes on the piano. When eleven he played and sang well. He finished grammar school when twelve years old, and when sixteen he completed the high school course.

"He was always truthful, honest, affectionate and generous, also very quick witted and kind to animals and little children.

"At about the age of fourteen, complaints began coming in rapidly. I was told that he took bicycles to pieces, overturned milk pitchers, grocers' wagons, sheds, etc. Then he began breaking into and pilfering from barns, Y. M. C. A. lockers, and stores. He was arrested a number of times, but set free on account of his youth and innocent appearance. The first time he was held by the police was for breaking into and robbing a store on Euclid Avenue. It was so skillfully done that the police could hardly believe it was the work of a boy. After the damages were paid he was released, only to do the same thing over and over again.

"At about this time he began to have violent spells, which usually came on in the early morning, during which he would walk about the house and rave, dragging his bedding from room to room. Sometimes he would see imaginary people and things. At one time he held his shoes in his hands and talked to them.

"At such times if I spoke to him, he would answer rationally and immediately begin again to rave. He would show me things which he thought he saw, and be impatient with me if I told him they were not there. At one time he took me up a flight of stairs to show me an imaginary steam boiler which he excitedly told me was about to burst. These spells would sometimes last for hours. This would have been recognized as insanity had not the police suggested that it was the effect of what they called 'dope.'

"After all that was possible was done for him, he was sentenced to the Ohio State Reformatory in the fall of 1905. He went willingly, saying that he realized that he could not control himself and that he hoped while there to be cured.

"He remained in the institution for two years and then came home sullen and with a feeling of contempt for all law and with a mania for knocking people down.

"In place of a laugh he had a sneer.

"When I wrote to you, in January, 1909, he was 'serving time' in the workhouse for assaulting a man. I knew nothing of your work, but on seeing a newspaper article which told me what you were doing, I immediately appealed to you as my last hope.

"After you had him operated upon, January 18, 1909, he began at once to improve. At first he had many and frequent relapses of extreme excitability and restlessness at night, but these spells came less often. Now he is sleeping quietly, is seldom irritable, laughs naturally, is cheerful and happy, has lost all desire to do wrong, and I am no longer urged to press the insanity charge which I had made against him, before I wrote to you."

In the course of the investigation of the younger inmates three older patients were seen incidentally, and operated on as a matter of faint hope, but without much expectation of a favorable result. The outcome, however, has been unexpectedly favorable in two of the cases. No report has been received of the other one.

One patient, Tom M., had been committed to the institution ninety-three times during the last thirty years for drunkenness and fighting. Although liquor always made him obstreperous, he was when sober, very anxious to stop drinking, and was pleasant and capable. Skiagraphic examination showed abscesses at the roots of two old stumps of teeth. These were extracted in February, 1909. Tom has since told me that before the extraction he had some tooth-ache, severe pain in the head, and sleeplessness, and in addition a periodic craving for drink which neither he nor anyone else had ever connected with the dental manifestations. At the last report he had been entirely sober and working faithfully for fifteen months.

The next patient, a women forty years old, was also unusually capable and faithful in her sober moments. She had, however, been committed twenty or thirty times in the last ten years for drunkenness. She had been sleepless, had a good deal of headache in the temples and the back of the head, and had suffered with terrible toothaches for several years past. The drinking had in her case never been associated by anybody with her bad teeth. She was well nourished and of fairly good color. She was very anxious to recover. On inspection the nineteen remaining teeth in her jaws were all found to be badly decayed and the gums deeply ulcerated. They were extracted. Following their extraction she went on one more spree, but at the last report had been entirely sober and at work for nine months.

These two patients appear to differ from the first one in being cases of periodic aberration caused by pain. There is no evidence, however, to show that the craving for drink is in such cases really due to any pain recognized as such in consciousness, or localized in any way. It is rather the result of vague but intense emotion, in the form of either unrest with depression, or elation with its accompanying lack of self-control.

In all three of these cases recovery seems to have been facilitated by an unusual original endowment both moral and mental. Such mentalities unless completely shattered may return to a condition of stable equilibrium on the removal of even a long-continued irritant cause of aberration. The chance of recovery is of course much better in early cases.—*Psychological Clinic.*

